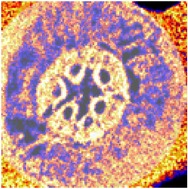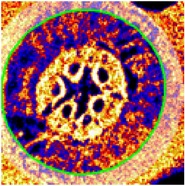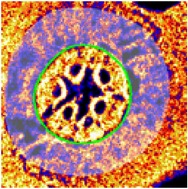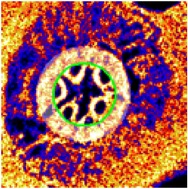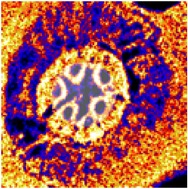# The Review of Nuclear Microscopy Techniques: An Approach for Nondestructive Trace Elemental Analysis and Mapping of Biological Materials

**DOI:** 10.1155/2015/740751

**Published:** 2015-11-18

**Authors:** Stephen Juma Mulware

**Affiliations:** Louisiana Accelerator Center, Department of Physics, University of Louisiana at Lafayette, Broussard Hall No. 115, Lafayette, LA 70504, USA

## Abstract

The properties of many biological materials often depend on the spatial distribution and concentration of the trace elements present in a matrix. Scientists have over the years tried various techniques including classical physical and chemical analyzing techniques each with relative level of accuracy. However, with the development of spatially sensitive submicron beams, the nuclear microprobe techniques using focused proton beams for the elemental analysis of biological materials have yielded significant success. In this paper, the basic principles of the commonly used microprobe techniques of STIM, RBS, and PIXE for trace elemental analysis are discussed. The details for sample preparation, the detection, and data collection and analysis are discussed. Finally, an application of the techniques to analysis of corn roots for elemental distribution and concentration is presented.

## 1. Introduction

The study of biological processes in living organisms shows that many important functions depend on the presence of specific essential/trace elements. The essential trace elements can be defined in simplest form as that element which is required in small quantities for the maintenance of life. Its absence or excessive presence beyond the right amounts results in either death or malfunctioning of specific organ in the living organism.

Out of all the naturally occurring elements, about 17 and 26 are known to be essential to plant life and animal life, respectively. For an element to be essential for plant growth, it must meet two main criteria in accordance with Liebig's law of minimum, a principle developed in agricultural sciences by Carl Sprengel (1828) and later popularized by Justus von Liebig, which states that growth of a plant is controlled not by the total amount of resources available but by the scarcest resource [1]. The two criteria are as follows: (1) in its absence the plant is unable to complete a normal life cycle or (2) the element is part of some essential plant constituent or metabolite. Apart from carbon and oxygen that are absorbed from the air and water which is absorbed from the soil, plants must also obtain the following mineral nutrients from the growing media: the primary macronutrients: nitrogen (N), phosphorus (P), and potassium (K); the three secondary macronutrients: calcium (Ca), sulphur (S), and magnesium (Mg); the macronutrient silicon (Si); and the micronutrients/trace minerals: boron (B), chlorine (Cl), manganese (Mn), iron (Fe), zinc (Zn), copper (Cu), molybdenum (Mo), nickel (Ni), selenium (Se), and sodium (Na). Amongst the 26 essential elements for animals, trace elements include iron (Fe), copper (Cu), zinc (Zn), manganese (Mn), cobalt (Co), nickel (Ni), molybdenum (Mo), Chromium (Cr), selenium (Se), iodine (I), fluorine (F), tin (Sn) silicon (Si), vanadium (V), and arsenic (As). The other 11, namely, carbon (C), hydrogen (H), oxygen (O), nitrogen (N), sulfur (S), calcium (Ca), phosphorous (P), sodium (Na), chlorine (Cl), potassium (K), and magnesium (Mg), are major elements for biological processes in systems [2].

The significance of the essential trace metals in biological systems is indisputable due to their positive roles when in specific concentration ranges and toxic roles in relatively high or low concentration levels. The essential trace metals have four main functions which include (i) stabilizers, (ii) elements of structure, (iii) essential elements for hormonal function, and (iv) cofactors in enzymes. Inadequacy or lack of trace elements will affect the structure alone or will affect structural function due to lack of stabilization, change of charge properties, and allosteric configuration [3]. An excess or imbalance of these elements has been implicated in the pathogenesis of several diseases like cancer, Parkinson's disease, and atherosclerosis in animals as well as phytotoxic effects in plants. Therefore, the development of techniques which can accurately map and measure trace elements has been a prime goal of scientists in the recent years. Nuclear microscopy employs a variety of high energy (MeV) ion beam techniques at submicron spatial resolutions to provide elemental imaging and quantitative elemental analysis of biological tissue down to the parts per million (ppm) level of analytical sensitivity. Nuclear microscopy is a focused MeV ion beam based group of techniques that has the capacity to image density variations in relatively thick or thin tissues/samples, map trace elements at the cellular level to the microgram per gram (dry weight) level, and extract quantitative information on these elements [4]. The nuclear microscopy constitutes a group of imaging and analytical techniques composed of scanning transmission ion microscopy (STIM), particle induced X-ray emission (PIXE), and Rutherford backscattering (RBS) which uses focused MeV proton beam in a microprobe for analysis.

## 2. Methods

### 2.1. Scanning Transmission Ion Microscopy (STIM)

STIM is used to identify regions of interest in the sample. STIM is often used to investigate samples thin enough to transmit a 2-3 MeV proton beam. For thin organic samples (30 *μ*m or less), essentially all incident protons which are not backscattered will be transmitted. STIM images are generated by measuring either the transmitted ion energy or variations in the number of ions at each image pixel within the scanned area. Only one ion per pixel is required in principle to measure the energy loss, but, in practice, owing to energy straggling, several ions are required to generate low noise images. The best method of generating STIM image is using an event-by-event data acquisition system. The measured data set of the ion energy loss values at each pixel can then be manipulated in different ways to give the best image contrast. With average processing, the outlying events in the energy spectrum at each pixel, arising from slit scattering or other forms of noise, have a large effect on the average energy loss and so can introduce noise into the image. This can be avoided by discarding the outlying events. With median processing, the measured energy loss values in each pixel are ordered by increasing energy loss, and the central value is chosen. This defines the location of edges present within a pixel and effectively eliminates noise in the image. The density structure of the sample can thus be determined by measuring the energy loss of the transmitted beam particles [5].

With STIM, the transmitted ion energies and number of ions at each pixel within the scanned area are measured using a semiconductor detector located behind the sample and used to generate an image showing variations in areal density. Scanning transmission ion microscopy was developed primarily as a method of quantitatively imaging the areal density distribution of thin biological samples and identifying features of interest for subsequent analysis with PIXE or backscattering spectrometry. It has also been used to normalize PIXE images to the measured variation of the sample areal density and to image the density of small living insects in air. An important feature of biomedical research is that STIM can image variations in the areal density of unstained tissue sections for subsequent PIXE analysis, thus avoiding the serious problem of contamination of samples with the chemical dyes that are normally used to highlight features.

### 2.2. Rutherford Backscattering Spectroscopy (RBS)

**Figure 1:**
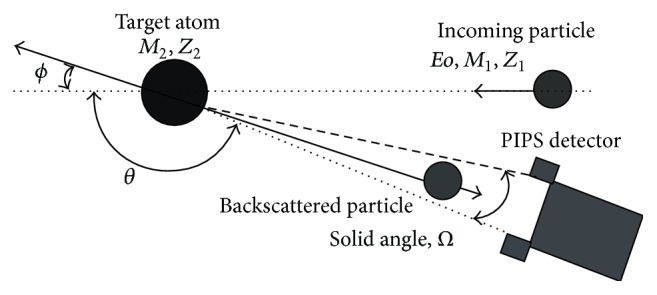
Elastic collision and typical geometry of RBS.

RBS is one of the most powerful techniques for measuring elemental depth profiles. With backscattering spectroscopy, the number and energy of elastically backscattered ions, usually H or He ions with energies in the range 1–4 MeV, are measured to determine the sample stoichiometry and elemental depth distributions. RBS is especially suitable for analysis of biomaterials for matrix composition because the bulk elements composition of such materials are C, N, and O whose spectrum plot edges clearly rise above the background. This enables easy fitting of the spectrum using non-Rutherford cross sections. Trace elements in biomaterials are very low in concentration and so the spectrum height at the appropriate energy edge given by *KEo* where *K* is the kinematic factor of the element is very small for significant analysis. This explains why RBS is not suited to measure trace element concentrations in biomaterials.  Figure 1 shows an elastic collision and typical geometry of RBS analysis.

For a given backscattering angle, nuclei of different elements in the sample scatter incident ions with different energies, producing separate peaks on a plot of counts versus energy. The spectrum edges are characteristic of the elements contained in the sample, providing a means of analyzing the composition of a sample by fitting the spectrum with known scattering cross sections [6].

### 2.3. PIXE Process

**Figure 2:**
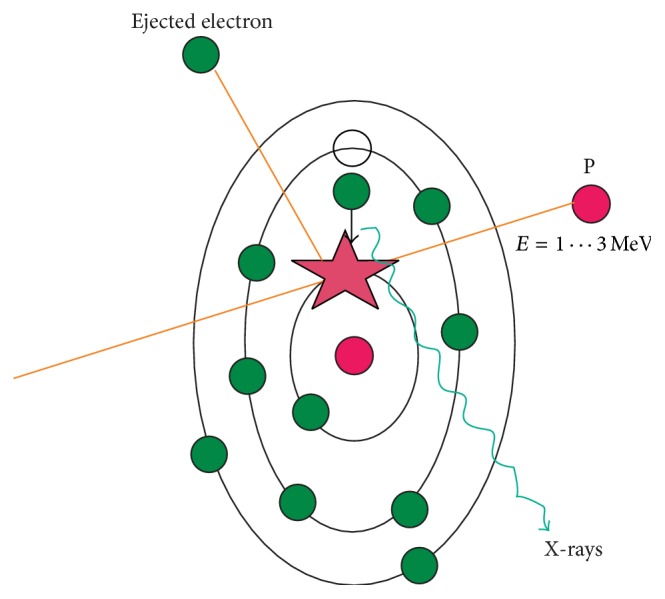
Ion projectile interaction with target during PIXE.

PIXE is a nondestructive technique that allows simultaneous multitrace elemental analysis down to the parts per million (ppm) levels. PIXE is based on the ejection of an inner shell electron out of its orbit by an interaction with MeV ion. As the vacancy is filled by an outer shell electron, an element and shell-specific X-ray is emitted. The energies of the X-rays are characteristic of the elements in the sample and the intensities of the X-rays can be used to calculate the abundance or concentration of an element. It is the most commonly used microprobe technique and has been widely applied in trace element analysis in biomedical and geological fields [7].  Figure 2 shows an ion projectile interaction with a target atom during PIXE.

When a typical MeV ion strikes a sample atom, the interaction generates a vacancy in the inner shells of the atom. MeV light ions have high cross section for ejecting K, L, or M shell electrons because their velocities approach the inner shell electron velocity. An inner vacancy exists for about 10^−7^ seconds before being filled by an electron transition from an outer shell with a subsequent emission of either X-ray or an auger electron. The energy of the emitted X-rays is unique to the element, so the measured X-ray energy of the spectrum allows the elements present in the sample to be identified. With PIXE, the measured X-ray yield is nearly independent of the chemical state or bonding within the sample and the X-ray production cross sections are well known; therefore, trace element concentration of less than 1 ppm can be detected and quantified [8].

The X-ray signal from the trace elements of interest can be partially or totally masked by an unwanted background in the spectrum arising from the backing or host material. This background can be in form of a continuum due to bremsstrahlung or it may contain discrete peaks arising from interfering characteristic X-ray from the matrix material. The decrease in the continuum at the lowest energies is due to absorption of X-rays in the Be window of the X-ray detector. The background at low energies prevents easy and precise analysis of light elements in the spectrum. This is why PIXE is often used for measuring trace elements of mass above sodium.

## 3. Experimental Considerations

### 3.1. The Charge Measurement

The ion beam intensity necessary for the quantitative analysis and normalization between sequential irradiations relies on accurate charge measurements. The current integration which is used to obtain charge can be determined by measuring the current on the conducting target or through the target on a faraday cup for thin targets. In case of insulating target, the current can be measured by evaporating or coating a thin layer of conducting carbon layer on the front surface of the target or by mixing a conducting powder with the target material in a predetermined quantity. The chamber is always biased appropriately to avoid the effect of secondary electrons on the charge measurements.

### 3.2. The X-Ray Detector

**Figure 3:**
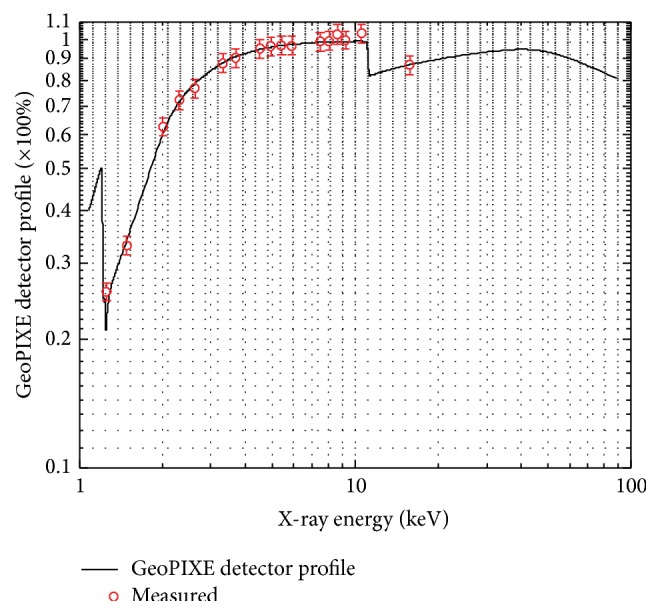
HPGe-detector efficiency calibration.

The High Purity Germanium- (HPGe-) detector was acquired for PIXE analysis of biological samples. The HPGe-detector was manufactured by Canberra Electronics and it is an ultralow energy detector model, GUL0110, that came equipped with ITRP type preamplifier. For absolute quantitative analysis to be performed, the detector efficiency must be accurately known over a given energy range large enough to encompass all typical characteristic X-rays of interest in the sample [9]. With the solid angle set at 205 msr, the detector was used for PIXE measurements of thick standard samples in order to calibrate it. A 2 MeV proton beam was used to excite X-rays in thick standard samples (acquired from Geller Microanalytical Laboratory, Inc.) of single and multielement composition of energy range from 1.25 keV to 25 keV. Simultaneous PIXE and RBS measurements were taken. A 75 *μ*m polyethylene filter was interposed in front of the detector to stop the backscattered protons. The detector profile was set up in GeoPIXE and the corresponding detector efficiency measured as shown in Figure 3 [10].

## 4. Application: Trace Elemental Mapping and Distribution of the Roots of Corn Seedling

The seeds of corn were germinated in vitro in the dark for a period of six days in agarose gel. The root was excised 5 mm long and inserted in a plastic tube filled with freezing medium and then quickly cryofrozen in a container of isopentane (2-methylbutane) cooled with liquid nitrogen, which provides superior cryogenic condition without Leidenfrost phenomenon (boiling of liquid nitrogen). The root was then cryosectioned 60 *μ*m thick and quickly freeze-dried. The samples were then irradiated with a 2 MeV focused proton beam of resolution of 5 *μ*m. Simultaneous RBS and PIXE were done and data collected. The RBS data was analyzed by SIMNRA [11] to determine the areal density and corrected charge which was then used to analyze the PIXE spectrum in GeoPIXE [12].

The GeoPIXE program used for the analysis in this project has an inbuilt ability to extract the X-ray yields from the spectrum via peak fitting. The yield *Y*_*k*_ calculations include absorption and secondary fluorescence contributions. The result is a standard less quantitative method with minimum detection limits down to 1 ppm level. For the elemental maps and concentration analysis, a dynamic analysis (DA) matrix of the fitted PIXE spectrum was generated and used to generate the elemental maps. The spectrum peak areas *a*_*k*_ are related to element concentration *C*_*k*_ by the following equation:

(1) $$\begin{array}{c} a_{k}=Q\Omega \varepsilon_{k}T_{k}Y_{k}C_{k}, \end{array}$$

where *Q* is the integrated beam charge, *Ω* and *ε*_*k*_ are the detector solid angle and efficiency, *T*_*k*_ is the X-ray absorber attenuation, and *Y*_*k*_ is the generic X-ray yield (counts per ppm *μ*mC for an ideal detector). *Y*_*k*_ is assumed to be constant for element *k* across the entire image. The solution of the linear least-squares problem can be cast as a matrix equation that transforms directly from spectrum represented by a vector *S* to concentration vector *C*, which includes all detected elements in terms of the matrix Γ [13, 14]:

(2) $$\begin{array}{c} C=Q^{-1}\Gamma S, \end{array}$$

where

(3) $$\begin{array}{c} \Gamma_{ki}=\left(\;\Omega \varepsilon_{k}T_{k}Y_{k}\;\right)\sum_{j}\alpha_{kj}^{-1}\beta_{ji}. \end{array}$$

**Figure 4:**
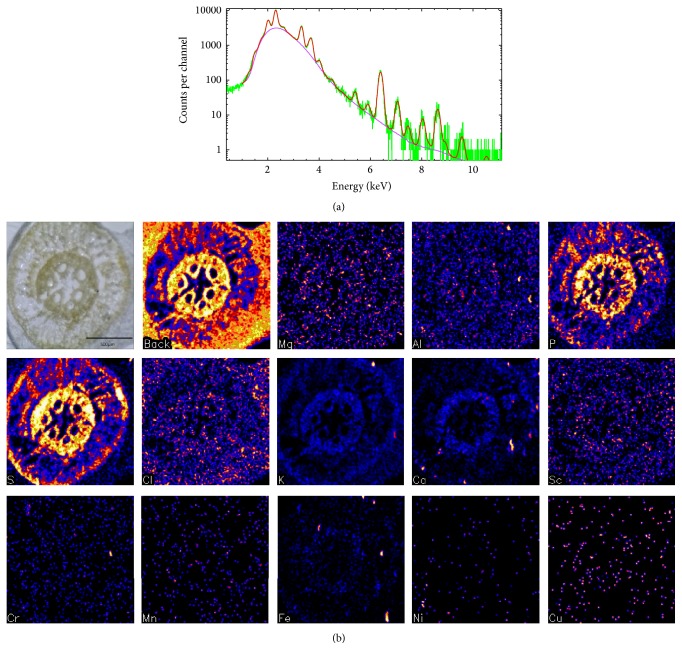
(a) A typical fitted PIXE spectrum of corn roots, (b) the corresponding elemental maps. The maps are as follows: top row: optical image, all elements-back image, Mg, Al, and P; second row: S, Cl, K, Ca, and Sc; bottom row: Cr, Mn, Fe, Ni, and Cu.

Usually, the Γ matrix is calculated in final linear least-squares iteration in the program PIXE-FIT, part of the GeoPIXE software package after all nonlinear parameters have converged and are fixed. The model function includes a complete set of X-ray lines for each element, detection artifacts (including tails, pile-up Ge escape peaks), and the SNIP background approximation corrected for absorption and detector efficiency [14]. The use of GeoPIXE software has been effective as demonstrated by the results of the analysis done by other plant scientists who analyzed the roots of a Mediterranean-type* Agathosma betulina* (Berg.) Pillans, colonized by* Cryptococcus laurentii* [15]. The micro-PIXE analysis revealed the presence of P, S, Cl, K, Ca, Ti, Cr, Mn, Fe, Ni, Cu, Zn, and As.  Figure 4 shows the fitted PIXE spectrum of the corn root and the corresponding elemental maps for the main elements detected in the root.

The average concentrations of the elements in the whole sections of the root were determined. The distribution of the elements in different regions of interest (ROI) of the root, whole root, the epidermis, the cortex, the endodermis, and the vascular tissue, was also determined and the 95% confidence interval of the average elemental concentrations calculated. The standard deviation of the mean for the elemental concentrations was also determined. The results were recorded as shown in Table 1.

## 5. Discussion

The distribution and quantification of trace elements in different sections of the corn root were successfully determined. The preferred accumulation sites and the highest levels of P, S, K, and Fe were detected in the epidermis and endodermis sections of the root. We also noticed reduced concentration of these elements in the cortex and vascular tissue sections of the corn roots. The elemental concentration of the other detected elements including Cl, Ca, Ti, Cr, Mn, Ni, Cu, Zn, and As was generally low. This could be attributed to low statistics or low absorption of these elements by the corn roots. Their distributions were much similar to the more abundant groups of P, S, K, and Fe discussed earlier. Similar observations were made by Cloete et al. in their study of elemental distribution and mapping of roots of a Mediterranean-type* Sclerophyll betulina* (Berg.) Pillans [15], in which they reported higher concentration of P and Fe in the epi/exodermal-outer cortical root tissues. They however reported a depletion of P and Fe in the endodermis and vascular tissues of the roots they studied. Their study also reported low concentration of the Mn, Cu, and Zn as well as Si, Cl, and K and attributed it to low statistics.

Nearly all the elements detected were primarily localized close to the symbiont exchange interface and within the epidermal and endodermal root tissues. This distribution pattern may be attributed to the presence of the apoplastic barrier present in the endodermis and the epidermis cell walls of root tissues, which tend to restrict the intertissue transport of certain elements [15]. However, it is important to note that the permeability of these apoplastic barriers cannot be reduced to zero, hence allowing some elements to pass through [16]. It is also important to note that symplastic transport could have occurred which led to low concentration of some elements in the outer epidermis compared to the endodermis sections of the roots [16–18].

For this biological sample of corn root, the nuclear microscopy was successfully used to map and quantitatively measure the elemental concentrations and distribution within the sample. This technique has proved to yield reliable results that can be extended to the analysis of other biological tissues. PIXE analysis consists of two parts as demonstrated. The first part is to identify the atomic species in the target from the energies of the characteristic peaks in the X-ray emission spectrum as shown in Figure 4. By using GeoPIXE features, the corresponding elemental maps were also generated. The second part is to determine the quantity of the element present in the target from the intensity of its characteristic X-ray emission spectrum. As shown in Table 1, it is also possible to analyze the distribution of the elemental concentrations in different regions of interest in the target sample.

## 6. Conclusion

The use of nuclear microscopy techniques shows a proven analytical tool that is competitive with other classical methods like inductively coupled plasma mass spectrometry (ICP-MS) [19] and atomic absorption spectrophotometer [20] that may have limited capability of mapping trace elements in biological materials. The inductively coupled plasma mass spectrometry (ICP-MS) is a type of mass spectroscopy that can detect metals and several nonmetals at concentrations as low as one part in 10^15^ (part per quadrillion, ppq) on noninterfered low-background isotopes. This is achieved by ionizing the sample with inductively coupled plasma and then using a mass spectrometer to separate and quantify those ions. Compared to atomic absorption techniques, which is an electroanalytical procedure for the quantitative determination of chemical elements using the absorption of optical radiation (light) by free atoms in the gaseous state, ICP-MS has greater speed, precision, and sensitivity. However, compared with other types of mass spectrometry, such as thermal ionization mass spectrometry (TIMS) and Glow Discharge Mass Spectrometry (GD-MS), ICP-MS introduces a lot of interfering species: argon from the plasma, component gasses of air that leak through the cone orifices, and contamination from glassware and the cones. The nuclear microscopy techniques are capable of multielemental mapping and analysis of biological materials as demonstrated in this work. A large number of elements can be mapped and quantitatively analyzed simultaneously and with the help of a suitable software like GeoPIXE, the elemental distribution within the sample can be accurately determined. The technique is sensitive enough and has a minimum detection limit down to 1 ppm and even lower. This tool is also nondestructive making it uniquely suitable for biological analysis.

## Figures and Tables

**Table 1:** The elemental concentrations in regions of interest (ROI) for a typical corn root sample. The maps below the table show the ROI: whole root, epidermis, cortex, endodermis, and vascular tissues. For each ROI, the average concentration, the 95% confidence interval, and the standard deviation are presented for all quantified elements.

Element	ROI whole			ROI epidermis			ROI cortex			ROI endodermis			ROI vascular tissues		
	Conc. ppm	CI 95%	SD	Conc. ppm	CI 95%	SD	Conc. ppm	CI 95%	SD	Conc. ppm	CI 95%	SD	Conc. ppm	CI 95%	SD
P	541	131	106	601	129	104	384	141	114	842	251	202	406	237	191
S	591	180	145	653	146	117	457	164	132	834	138	111	312	57	46
Cl	18	12	9	34	28	23	11	9	7	21	18	15	4	10	8
K	163	131	106	177	118	95	150	137	110	205	160	128	153	127	102
Ca	52	46	37	66	66	53	31	7	6	60	39	31	23	4	3
Ti	11	17	13	18	28	22	10	16	13	20	34	28	9	17	13
Cr	2.3	1	1	2	1	1	2	1	1	4	4	3	3	2	2
Mn	1.3	1	1	1	2	2	1	1	1	2	1	1	3	3	2
Fe	106	27	22	114	23	18	85	30	24	129	28	22	67	32	26
Ni	2.8	5	4	2	4	3	4	6	5	1	2	1	12	24	19
Cu	3.2	2	1	2	2	2	4	4	3	1	2	1	13	20	16
Zn	7.7	6	5	4	4	3	11	11	9	5	8	6	16	24	19
As	3.1	2	2	2	3	3	9	14	11	1	3	2	11	18	14